# Haptic-Extended Flange Intrascleral Fixation for Single-Piece Acrylic Intraocular Lenses

**DOI:** 10.3390/jcm15166190

**Published:** 2026-08-10

**Authors:** Ayako Eno, Kuo-Chung Chang, Tetsuro Oshika

**Affiliations:** 1 Eno Eye Clinic NishiAshiya, Ashiya 659-0085, Hyogo, Japan; aeno@enoganka.com (A.E.); kokuchu.cho668@gmail.com (K.-C.C.); 2 Faculty of Medicine, University of Tsukuba, 1-1-1 Tennoudai, Tsukuba 305-8575, Ibaraki, Japan

**Keywords:** single-piece IOL, intrascleral fixation, tilt, decentration, presbyopia-correcting IOL

## Abstract

**Background/Objective**: Whereas conventional flanged intrascleral fixation is limited to 3-piece intraocular lenses (IOLs), we developed and evaluated a novel haptic-extended flange technique for single-piece acrylic IOLs, including presbyopia-correcting and toric lenses. **Methods**: In this technique, a 6-0 polypropylene suture is secured along the lateral aspect of the single-piece IOL haptic to extend its length, followed by intrascleral flange fixation. Uncorrected visual acuity (UCVA) from far to near, corrected distance visual acuity (CDVA), and refraction were evaluated at 1 month postoperatively. IOL tilt and decentration were assessed using anterior segment optical coherence tomography. **Results**: This study included a consecutive series of 10 eyes of 10 patients (mean age: 58.3 ± 16.2 years), comprising 5 eyes with multifocal IOL, 2 eyes with multifocal toric IOL, 2 eyes with enhanced monofocal IOL, and 1 eye with monofocal IOL. All IOLs were securely fixed; the mean IOL tilt was 5.74 ± 2.93° and the mean decentration was 0.35 ± 0.19 mm. All eyes achieved CDVA of 20/20 or better, and all eyes with multifocal IOLs achieved UCVA at 40 cm of 20/25 or better. No early postoperative complications attributable to the surgical procedure were observed. In a case associated with atopic dermatitis, retinal detachment occurred 4 months after surgery. **Conclusions**: In this preliminary case series, haptic-extended flange fixation enabled early stabilization of selected single-piece acrylic IOLs with favorable short-term visual and anatomic outcomes. Larger studies with longer follow-up are needed to confirm long-term safety, durability and comparative effectiveness.

## 1. Introduction

Numerous surgical techniques have been described for intraocular lens (IOL) fixation in eyes with inadequate or absent capsular support. Among these, the Yamane flanged intrascleral haptic fixation technique has gained widespread adoption owing to its relative simplicity, minimal invasiveness, and favorable clinical outcomes, including excellent IOL stability with minimal tilt and decentration [1]. However, as this technique is specifically designed for 3-piece IOLs, it cannot be applied to single-piece platforms, which currently serve as the optical basis for most contemporary presbyopia-correcting and toric IOLs. When a single-piece IOL is explanted and replaced with a 3-piece IOL via intrascleral fixation due to compromised capsular support, the premium features of the original lens are lost, resulting in postoperative visual outcomes that fall far short of patient expectations. Furthermore, exchanging the decentered IOL for a new one, rather than repositioning the existing lens, imposes significant surgical trauma on the eye.

To achieve scleral fixation of single-piece IOLs, several approaches have been proposed, including piercing the optic or haptics for suture fixation [2,3,4,5] and tying sutures directly to the haptics for 2-point fixation [6,7,8]. Nevertheless, each of these methods carries notable limitations. Piercing procedures are technically demanding, risk compromising the structural integrity of the IOL, and often necessitate temporary lens externalization, in some cases requiring a specialized instrument such as an IOL punch. Haptic-tying suture methods involve complex intraocular manipulation and carry an inherent risk of postoperative IOL tilt attributable to the geometric instability of 2-point fixation.

In the Yamane flanged intrascleral fixation technique for 3-piece IOLs, an intrascleral tunnel of roughly 2 mm provides a long contact surface between the haptic and scleral tissue, which increases friction and mechanical interlock. This reduces axial shift and IOL tilt compared with very short entries, contributing to a more predictable effective lens position and stable postoperative refraction [1]. To extend these clinical and mechanical advantages to the fixation of single-piece IOLs, we developed a novel, haptic-extended flange fixation technique using a 6-0 polypropylene suture. In this approach, a 6-0 polypropylene suture is secured along the lateral aspect of the single-piece IOL haptic to augment its effective length, thereby enabling intrascleral flange fixation. Similar to 3-piece IOL haptics, a 6-0 polypropylene suture can be used for intrascleral fixation by heat-treating its tip. Additionally, because the suture is secured to the haptics with multiple knots rather than at a single point, IOL decentration and tilt can be effectively prevented. The present study evaluates the clinical efficacy of this technique with respect to postoperative visual outcomes, refractive predictability, and IOL alignment, including tilt and decentration. We hypothesized that the haptic-extended flange fixation technique would provide acceptable IOL tilt and decentration while maintaining the optical performance of selected single-piece IOLs.

## 2. Materials and Methods

### 2.1. Patients

This single-center, retrospective, sequential interventional case series included consecutive cases who had received intrascleral fixation of a single-piece acrylic IOL due to insufficient capsular support between June 2023 and April 2026. All surgeries were performed by a single experienced surgeon. Clinical data were retrospectively extracted from medical records.

Inclusion criteria comprised eyes with decentration or dislocation of an existing single-piece acrylic IOL, as well as those in which a single-piece acrylic IOL was indicated despite inadequate or compromised capsular support. Exclusion criteria included active intraocular inflammation or infection, inadequate scleral or conjunctival integrity, uncontrolled glaucoma, severe endothelial compromise, or untreated vision-limiting posterior-segment disease. Additionally, eyes with trauma-related iris instability or those requiring complex combined surgery were considered high-risk and were excluded at the surgeon’s discretion.

The study protocol was reviewed and approved by the Tokushukai Group Central Ethics Committee (Approval Number: TGE02955-969). Although this study was retrospective in design, the Ethics Committee approved not only the retrospective collection of existing data but also the prospective acquisition of additional data following approval. Comprehensive study information was provided to all participants, and written informed consent was obtained from each individual. The study adhered to the tenets of the Declaration of Helsinki and was conducted in accordance with the Ethical Guidelines for Medical and Health Research Involving Human Subjects in Japan.

During the preparation of this manuscript, generative AI (Gemini 3.6 Flash) was used solely for the purpose of checking and refining English grammar. The tool was not utilized for any other tasks, including study design, data collection, or data analysis.

### 2.2. Surgical Technique

**Figure 1:**
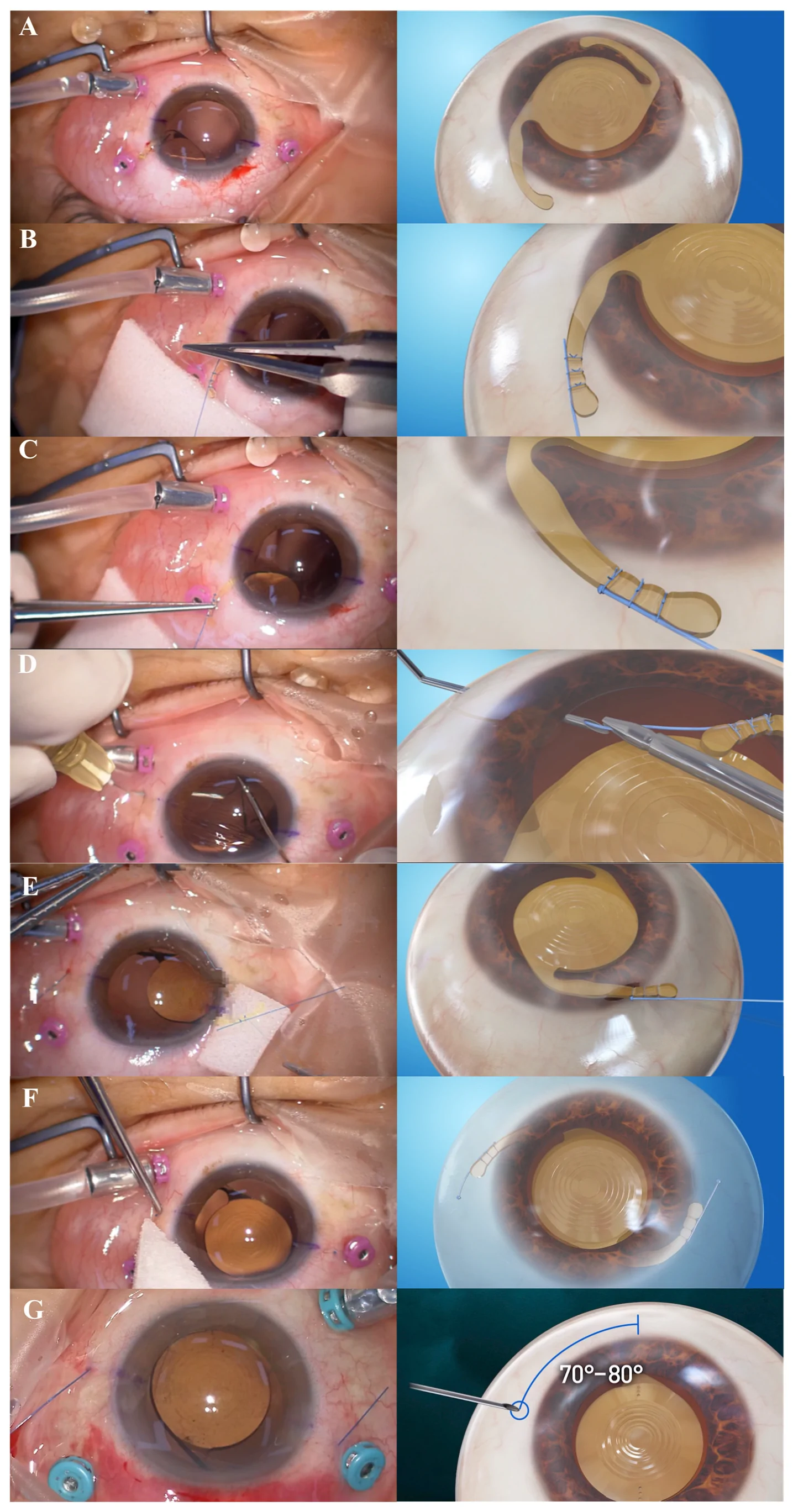
Surgical steps of the haptic-extended flange fixation technique. (**A**): Exteriorization of the IOL haptic through a corneal limbal side-port incision. (**B**): Alignment of 6-0 polypropylene suture parallel along the lateral side of the IOL haptic, secured with 3 knots using 10-0 polypropylene sutures. (**C**): Creation of a flange at the optic-side terminal of the 6-0 polypropylene suture using a handheld cautery device to prevent slippage. (**D**): Internalization of the haptic-suture complex, with the free end of the 6-0 polypropylene suture threaded into a 30-gauge needle for externalization. (**E**): Replication of the same suture-extension procedure on the contralateral haptic. (**F**): Creation of terminal flanges at both suture ends to achieve stable and secure intrascleral fixation. (**G**): For toric IOLs, insertion of the 30-gauge needles at locations shifted 70° to 80° away from the intended alignment axis, followed by the same fixation procedure.

The presence or absence of retinal abnormalities was assessed by preoperative fundus examination and intraoperative fundus visualization during vitrectomy. Following pars plana vitrectomy, the IOL haptic was exteriorized through a corneal limbal side-port incision (Figure 1A). An 11-mm length of 6-0 polypropylene suture (MANI, Inc., Tochigi, Japan) was aligned along the lateral side of the IOL haptic and secured with 3 knots using 10-0 polypropylene sutures (MANI, Inc.) (Figure 1B). It is critical to secure the 6-0 polypropylene suture strictly parallel to the IOL haptic. Heat was applied to the optic-side terminal of the 6-0 polypropylene suture using a handheld ophthalmic cautery device (Accu-Temp Cautery, Beaver Visitec International, Waltham, MA, USA) to create a flange, which prevented the suture from slipping off (Figure 1C). Subsequently, the haptic-suture complex was internalized into the eye. Following the Yamane technique, the free end of the 6-0 polypropylene suture was threaded into a 30-gauge needle (Figure 1D) and externalized. The same procedure was performed on the contralateral haptic (Figure 1E), and flanges were created at both suture ends to achieve secure intrascleral fixation (Figure 1F). The suture tip flange size was approximately 300 µm, with approximately 1.5–2.0 mm of haptic melted. The flange was slightly larger than the needle outer diameter but not excessively large, to avoid exposure or disinsertion. The flange was buried within the sclera. The surgical technique is demonstrated in Supplementary Videos S1–S6.

For toric IOLs, the 30-gauge needles were inserted at locations shifted 70° to 80° away from the intended alignment axis, and the same fixation procedure was performed (Figure 1G).

### 2.3. Measurements

Monocular corrected distance visual acuity (CDVA) at 5 m was measured at 1 month postoperatively in all eyes. For eyes implanted with multifocal IOLs, monocular uncorrected visual acuity (UCVA) was evaluated at multiple distances (5 m, 3 m, 1 m, 60 cm, and 40 cm). Subjective spherical equivalent was calculated for all eyes except one that was targeted for −3.0 D. Visual acuity and refraction were assessed under standardized photopic conditions. The examiners were not masked to the patients’ clinical information.

IOL tilt and decentration were evaluated at 1 month postoperatively in all eyes using anterior segment optical coherence tomography (AS-OCT; CASIA2, Tomey, Aichi, Japan). This device uses a 1310-nm swept-source laser wavelength and acquires images in 0.3 s, producing 16 AS-OCT images from 16 different meridians for three-dimensional analysis. Unlike conventional two-dimensional measurements limited to vertical and horizontal axes, this system enables three-dimensional assessment of IOL tilt. For AS-OCT imaging, patients fixated on the internal target, and a minimum of 2 measurements were taken to confirm stability and reproducibility. CASIA2 measures decentration and tilt using the corneal topographic axis (also termed the corneal vertex normal) as the reference. This reference line connects the fixation point to the corneal vertex and is defined as the normal to the cornea at the vertex. No predetermined pupil size was required for these measurements, as pupil dilation has no impact on the results.

### 2.4. Statistical Analysis

Because this was an exploratory study with a small sample size, it did not have adequate statistical power for inferential analyses; therefore, only descriptive statistics are reported. Quantitative data are expressed as the mean ± standard deviation. Visual acuity values were converted into the logarithmic minimum angle of resolution (logMAR) for arithmetic averaging.

## 3. Results

Ten eyes of ten patients (mean age 58.3 ± 16.2 years) were included in the subjects, comprising 4 eyes with the Clareon PanOptix trifocal IOL (CNWTT0, Alcon Vision LLC, Fort Worth, TX, USA), 2 eyes with the Clareon PanOptix trifocal toric IOL (CNWTTx, Alcon Vision LLC), 2 eyes with the Eyhance-enhanced monofocal IOL (DIB00V, Johnson & Johnson Vision, New Brunswick, NJ, USA), 1 eye with TECNIS Multifocal +3.75 D (ZMF375, Johnson & Johnson Vision), and 1 eye with AcrySof IQ (Alcon Vision LLC). The patients’ background data are shown in Table 1.

In eight of the ten cases, the surgery was indicated for the dislocation or subluxation of existing single-piece acrylic IOLs, all of which were successfully preserved and repositioned using this intrascleral fixation technique. In one case, significant posterior capsular rupture due to an intraoperative complication necessitated the use of this technique to fixate the trifocal IOL to the sclera. In another case, an IOL that had been implanted several years prior was explanted due to calcification-induced opacification, and a new trifocal IOL was intrasclerally fixated using this technique.

Postoperative outcomes are summarized in Table 2 and Table 3. No significant refractive errors occurred, and CDVA results were favorable. In the eyes with multifocal IOLs, excellent UCVA was achieved at all distances, from far to near. Additionally, IOL tilt and decentration measured using AS-OCT were found to be minimal. There was one outlier case showing a tilt of 13.2°. This was a case involving a PanOptix IOL, with CDVA of 1.2 (20/16), UCVA at 5 m of 1.0 (20/20), and UCVA at 40 cm of 0.9 (20/22). The contrast sensitivity was within the normal range, and there was no report of dysphotopsia or reduction in quality of vision.

The residual astigmatism in two eyes implanted with toric IOLs was −0.75 D and 0 D. However, the final axis and degree of rotation could not be retrieved.

No intraoperative or postoperative complications associated with this technique were observed during the 2-month follow-up period. Specifically, there were no cases of pigment dispersion, iris capture, uveitis, hyphema, elevated intraocular pressure (IOP), cystoid macular edema (CME), corneal endothelial cell loss, hypotony, endophthalmitis, chronic inflammation, or suture exposure. At 4 months postoperatively, one eye in a patient with atopic dermatitis developed retinal detachment due to an ora serrata tear, which was successfully treated with vitrectomy and intraocular gas tamponade using sulfur hexafluoride (SF_6_). At 1 month after vitrectomy, the IOL tilt was 4.9° and the decentration was 0.41 mm, demonstrating favorable IOL fixation.

## 4. Discussion

In the present study, IOL positioning was evaluated using AS-OCT (CASIA2) with 3-dimensional analysis rather than conventional 2-dimensional measurement in the vertical and horizontal planes alone. At 1 month postoperatively, the mean IOL tilt was 5.74 ± 2.93° and the mean decentration was 0.35 ± 0.19 mm. Comparable studies employing 3-dimensional assessment of IOL position following intrascleral fixation using the Yamane technique have reported varying alignment outcomes, with mean tilt and decentration values of 8.8 ± 3.9° and 0.52 ± 0.35 mm [9], 7.67 ± 3.70° and 0.57 ± 0.37 mm [10], and 2.31 ± 0.93° and 0.20 ± 0.13 mm [11], respectively. Furthermore, postoperative values ranging from 5.51 ± 3.17° to 6.33 ± 3.8° for tilt, and 0.46 ± 0.22 mm to 0.49 ± 0.24 mm for decentration, have also been described [12]. Although our proposed procedure was performed on single-piece IOLs and involved more complex surgical maneuvers compared with these for 3-piece IOLs, our clinical outcomes regarding postoperative IOL tilt and decentration were highly comparable to those established values.

Regarding the transscleral fixation of a single-piece IOL in the absence of capsular support, a previous case report evaluating a 2-point suture technique documented postoperative IOL tilt of 8.7° and decentration of 0.51 mm [7]. However, tilt and decentration were not evaluated in studies on piercing techniques for single-piece IOLs [2,3,4,5] or in other reports of 2-point suture fixation [6,8], a direct comparison with our findings remains precluded.

In our study, a case with atopic dermatitis developed retinal detachment secondary to a retinal tear at the ora serrata, which was successfully treated with pars plana vitrectomy and intraocular tamponade using SF_6_ gas. After the retinal detachment was resolved, the evaluated IOL positioning remained highly favorable, with a tilt of 4.9° and decentration of 0.41 mm. This finding suggests that the IOL secured by this technique maintains good stability, appearing capable of tolerating the physical stresses associated with vitreoretinal surgery and gas tamponade.

IOL tilt and decentration following routine cataract surgery have been more extensively investigated using 3-dimensional AS-OCT. A prospective clinical study employing a contemporary monofocal IOL (Clareon CNA0T0) reported a mean IOL tilt of approximately 5.3 ± 1.4° and a mean decentration of 0.27 ± 0.16 mm, with no clinically significant correlation with best-corrected distance visual acuity [13]. Another study has similarly documented typical postoperative IOL tilt in the range of approximately 2–5° and decentration of approximately 0.2–0.3 mm after standard cataract surgery [14]. Kimura et al. [15] observed a mean IOL tilt of 4.31° in the inferotemporal direction relative to the corneal topographic axis and a mean decentration of 0.05 mm temporally. Zhang et al. [16] reported that the ZMB00 IOL demonstrated mean decentration and tilt of 0.17 ± 0.11 mm and 4.84 ± 1.37°, respectively, while the ZXR00 IOL exhibited values of 0.18 ± 0.12 mm and 4.82 ± 1.37°. A widely cited theoretical and clinical analysis has suggested that decentration exceeding 1 mm and tilt greater than 5° may become visually significant by inducing oblique astigmatism and higher-order aberrations, particularly with aspheric and presbyopia-correcting IOLs [17]. Taken together, these findings suggest that the IOL alignment achieved with our haptic-extended flange fixation technique was clinically acceptable.

Regarding postoperative refractive and visual outcomes, as shown in Table 2, highly predictable results were achieved. The postoperative refraction was successfully aligned with the target of emmetropia in almost all cases. In terms of visual acuity, postoperative CDVA was excellent, reaching a mean of −0.08 logMAR. Furthermore, in the 7 cases where multifocal IOLs were utilized, patients achieved excellent UCVA at all distances, spanning far, intermediate, and near. The fact that the intended effect of a presbyopia-correcting IOL was sufficiently obtained despite the IOL not being fixated within the capsular bag suggests the potential utility of this technique. To further validate these postulates, future studies with larger patient cohorts, longer follow-up periods, and the inclusion of other premium IOL platforms are warranted.

Most contemporary premium IOLs, such as presbyopia-correcting and toric IOLs, are designed on single-piece platforms. When these IOLs become severely decentered or dislocated, repositioning them in situ using conventional techniques is generally not feasible. Consequently, the standard clinical approach has been IOL explantation followed by the secondary intrascleral fixation of a monofocal 3-piece IOL. In doing so, however, the presbyopia-correcting or astigmatism-correcting capabilities of the original premium lens are inevitably lost. Our novel technique overcomes these limitations by enabling the preservation and reuse of the dislocated single-piece IOL without requiring its explantation. By temporarily externalizing only the haptics through a side-port incision, the IOL can be successfully secured intrasclerally in situ. This approach is less invasive to the eye, minimizes damage to corneal endothelial cells, and avoids inducing additional corneal astigmatism. Furthermore, because it preserves and reuses the existing IOL rather than requiring exchange for a new lens, this technique may also offer advantages from a health-economic perspective. In theory, the current technique can reduce surgical invasiveness and material costs, but a formal cost-effectiveness analysis was outside the scope of this study.

The indications for our technique extend well beyond the repositioning of already dislocated or decentered IOLs. For instance, in cases complicated by extensive posterior capsular rupture or severe zonular dialysis during routine cataract surgery, utilizing this technique allows the surgeon to successfully proceed with the implantation of the preoperatively planned single-piece IOL. Furthermore, in cases where an IOL exchange is required for other clinical reasons, this method significantly expands the range of available IOL options, which represents another major advantage of this procedure.

The single most critical factor for achieving optimal surgical outcomes with this technique is securing the 6-0 polypropylene suture perfectly parallel to the IOL haptic. If the 6-0 polypropylene suture becomes oblique relative to the haptic, the risk of postoperative IOL tilt and decentration increases significantly. Therefore, meticulous alignment at this suture-haptic interface represents the most crucial aspect of the procedure. Aside from this specific modification, the remaining surgical steps do not differ substantially from the conventional Yamane technique. Threading the suture into the lumen of a 30-gauge needle is relatively straightforward because the 6-0 polypropylene suture is long and flexible, allowing its direction to be easily adjusted. In this respect, the maneuver may be easier than docking the rigid haptic of a 3-piece IOL into a 30-gauge needle, as required in the conventional Yamane technique. However, because the thin polypropylene suture can easily slip out of the needle lumen, particular care is required when externalizing it from the eye. Once the suture has been externalized, its generous length provides sufficient slack, thereby offering a wider safety margin and greater technical flexibility during fine adjustments of the final intraocular positioning of the IOL. Overall, we believe that surgeons with prior experience in the Yamane technique can master this method without major difficulties, and its learning curve is considered to be relatively short. Although the operative time varies significantly depending on the case, it generally ranges from 30 min to 1 h.

Regarding its indications, this technique is applicable specifically to single-piece IOLs with an open-loop haptic design, whereas those with closed-loop or plate-haptic configurations are excluded. While the clinical benefits are undoubtedly most prominent for premium IOLs, this approach is also highly advantageous for standard monofocal models. By allowing the original lens to be preserved and secured in situ, the procedure minimizes surgical invasiveness by eliminating the need for an IOL exchange.

The contraindications for this technique are essentially identical to those for the standard Yamane technique. Absolute contraindications to avoid or defer intrascleral IOL fixation include active intraocular inflammation or infection, inadequate scleral thickness or unhealthy/scarred conjunctiva at planned haptic-exit sites, uncontrolled glaucoma, severe endothelial compromise, untreated retinal or macular disease that limits visual potential, and the lack of a suitable flange-compatible IOL [18]. Furthermore, trauma-related floppy iris, marked iris instability, prior surgeries involving the conjunctiva (e.g., scleral buckling or glaucoma drainage device implantation), and the need for complex combined surgeries serve as relative contraindications, as they increase the risks of iris capture, uveitis-glaucoma-hyphema (UGH) syndrome, haptic erosion or extrusion, hypotony, intraocular pressure elevation, decentration, CME, and retinal detachment [19]. Controlled uveitis alone is not an absolute contraindication, but surgery should be undertaken only when the eye is completely quiet and accompanied by a rigorous inflammation-management plan [18].

This study has several limitations that should be acknowledged. First, this technique is applicable only to acrylic IOLs with an open C-loop haptic configuration. Second, compared with the original Yamane technique, a relative disadvantage is the requirement of 2 different types of suture materials. Third, all procedures were performed by a single surgeon, which may limit the generalizability of the results. Fourth, the retrospective design of this study introduces inherent selection bias and restricts the ability to draw causal inferences. Fifth, although postoperative slit-lamp examinations confirmed that all IOLs remained well-centered without iris chafing, iris capture, or pigment dispersion, detailed high-resolution imaging of the retro-iridial space (such as ultrasound biomicroscopy) was not performed. Consequently, exact millimeter-level measurements of the distances between the IOL optic or haptics, the ciliary sulcus, and the posterior iris surface were not obtained. Sixth, this study is constrained by its small sample size, relatively short postoperative follow-up period, and the absence of a comparative control group. Given that this surgical technique is performed only in a limited number of cases, recruiting and establishing a matching control group for comparison was not practically feasible. Future investigations involving larger cohorts, extended follow-up periods, and comprehensive evaluations of higher-order aberrations and patient-reported visual quality are warranted.

## 5. Conclusions

Despite the inherent technical challenges associated with the transscleral fixation of single-piece IOLs, our findings demonstrate that this novel technique yields excellent postoperative stability and highly favorable, precise IOL positioning in eyes lacking adequate capsular support.

## Figures and Tables

**Table 1:** Patient demographics.

Eyes/patients	10/10
Age, years	58.3 ± 16.2 (28, 78)
Female/male	4/6
Left/right	5/5
Axial length, mm	25.41 ± 1.36 (23.36, 27.49)
Preoperative CDVA, logMAR	0.00 ± 0.13 (−0.18, 0.30)
Preoperative UCVA at 5 m, logMAR	0.61 ± 0.78 (0.0, 2.0)

Mean ± standard deviation (range); CDVA = corrected distance visual acuity; logMAR = logarithm of minimum angle of resolution; UCVA = uncorrected visual acuity.

**Table 2:** Clinical outcomes at 1 month postoperatively.

	Multifocal IOL (*n* = 7)	All (*n* = 10)
Spherical equivalent, D	0.07 ± 0.26 (−0.25, 0.25)	0.18 ± 0.24 (−0.5, 0.25) *
CDVA, logMAR	−0.08 (−0.08, −0.08)	−0.08 ± 0.04 (−0.18, 0)
UCVA at 5 m, logMAR	−0.02 ± 0.06 (−0.08, 0.10)	-
UCVA at 3 m, logMAR	−0.06 ± 0.05 (−0.08, 0.05)	-
UCVA at 1 m, logMAR	0.04 ± 0.06 (0, 0.15)	-
UCVA at 60 cm, logMAR	0.02 ± 0.04 (0, 0.10)	-
UCVA at 40 cm, logMAR	0.02 ± 0.02 (0, 0.05)	-
Tilt, degrees	6.17 ± 3.32 (3.5, 13.2)	5.74 ± 2.93 (3.4, 13.2)
Decentration, mm	0.34 ± 0.21 (0.08, 0.7)	0.35 ± 0.19 (0.08, 0.7)

* *n* = 9 (one eye was excluded due to myopic target). Mean ± standard deviation (range); D = diopters; CDVA = corrected distance visual acuity; logMAR = logarithm of the minimum angle of resolution; UCVA = uncorrected visual acuity.

**Table 3:** Clinical data of 10 patients.

No	Eye	Age	Sex	IOL Model	Indication (Cataract/Comorbidity)	Axial Length (mm)	Endothelial Cell Density (/mm^2^)		Preop CDVA, logMAR	Postop CDVA, logMAR	Postop UCVA, logMAR					SE (D)	Tilt (°)	Decentration (mm)	Follow-Up (Months)
							Preop	Postop			5 m	3 m	1 m	60 cm	40 cm				
1	L	48	M	PanOptix	Traumatic cataract/IOL dislocation	NA	2786	2793	−0.176	−0.08	−0.08	−0.08	0	0	0	+0.25	3.5	0.08	14
2	R	43	M	PanOptix	Atopic cataract/IOL dislocation	27.01	2825	2760	0	−0.08	0.046	0.046	0.097	0	0	+0.25	4.9	0.41	37
3	L	78	F	Eyhance	Senile cataract with exfoliation/IOL dislocation	23.36	2294	2028	0	−0.08	0					+0.25	3.4	0.36	18
4	R	71	M	Eyhance	Senile cataract with exfoliation/IOL dislocation	27.49	1442	1450	0	0	1					−3.00	4.4	0.53	19
5	L	66	F	PanOptix toric (T3)	Senile cataract/intraoperative complication	26.00	2522	2544	0.301	−0.08	0.097	−0.08	0	0	0	+0.25	3.6	0.30	29
6	L	75	F	PanOptix	Senile cataract/IOL opacity (Lentis M + X)	25.21	2311	1835	0	−0.08	0	−0.08	0	0	0.046	−0.50	8.8	0.70	9
7	R	51	M	PanOptix toric (T4)	Atopic cataract/IOL dislocation	24.89	2892	2848	0	−0.08	0	−0.08	0	0	0.046	+0.25	13.2	0.52	9
8	R	53	M	PanOptix	Atopic cataract/IOL dislocation	24.62	2554	2430	−0.176	−0.08	−0.08	−0.08	0.046	0.046	0.046	0.00	4.2	0.27	6
9	L	28	M	TECNIS multifocal +3.75 D	Atopic cataract/IOL dislocation	24.70	3051	3124	−0.176	−0.08	−0.08	−0.08		0.097	0	0.00	5.0	0.08	2
10	R	70	M	AcrySof IQ	Senile cataract/IOL dislocation	NA	1277	1328	−0.176	−0.08	−0.08					0.00	6.4	0.20	2

IOL = intraocular lens; CDVA = corrected distance visual acuity; logMAR = logarithm of the minimum angle of resolution; UCVA = uncorrected visual acuity; D = diopters; SE = spherical equivalent.

## Data Availability

Data is registered at FigShare. https://doi.org/10.6084/m9.figshare.32917904 (accessed on 7 July 2026).

## Supplementary Materials

The following supporting information can be downloaded at https://www.mdpi.com/article/10.3390/jcm15166190/s1, Video S1: step 1A; Video S2: step 1B; Video S3: step 1C; Video S4: step 1D; Video S5: step 1E; Video S6: step 1F.

## Abbreviations

The following abbreviations are used in this manuscript:

IOL/IOLs	Intraocular lens/intraocular lenses
UCVA	Uncorrected visual acuity
CDVA	Corrected distance visual acuity
D	Diopters
logMAR	Logarithm of minimum angle of resolution
AS-OCT	Anterior segment optical coherence tomography
